# Application of Biosensors in Detecting Breast Cancer Metastasis

**DOI:** 10.3390/s23218813

**Published:** 2023-10-30

**Authors:** Yu Deng, Yubi Zhang, Meng Zhou, Bin Wu, Jing Zhou

**Affiliations:** 1 Department of Breast and Thyroid Surgery, Union Hospital, Tongji Medical College, Huazhong University of Science and Technology, Wuhan 430030, China; 2 Department of Orthopedics, Union Hospital, Tongji Medical College, Huazhong University of Science and Technology, Wuhan 430022, China; 3 Department of Breast and Thyroid Surgery, People’s Hospital of Dongxihu District Wuhan City and Union Dongxihu Hospital, Huazhong University of Science and Technology, Wuhan 430040, China

**Keywords:** breast cancer, metastasis, biomarker, biosensor

## Abstract

Breast cancer has garnered global attention due to its high incidence worldwide, and even more noteworthy is that approximately 90% deaths due to breast cancer are attributed to cancer metastasis. Therefore, the early diagnosis of breast cancer metastasis holds significant importance for reducing mortality outcomes. Biosensors play a crucial role in the early detection of metastatic breast cancer due to their advantages, such as ease of use, portability, and real-time analysis capabilities. This review primarily described various types of sensors for detecting breast cancer metastasis based on biomarkers and cell characteristics, including electrochemical, optical, and microfluidic chips. We offered detailed descriptions of the performance of these various biosensors and made comparisons between them. Furthermore, we described the pathology of breast cancer and summarized commonly used biomarkers for metastatic breast cancer. Finally, we discussed the advantages of current-stage biosensors and the challenges that need to be addressed, as well as prospects for their future development.

## 1. Introduction

Breast cancer has drawn global attention due to its high incidence worldwide. Based on data from the International Agency for Research on Cancer (IARC) in 2020, there were more than 2.26 million new cases of breast cancer worldwide, and nearly 685,000 people died from breast cancer, accounting for 15.5% of the total female cancer deaths globally, making it the most common cause of female cancer-related deaths globally [1]. Breast cancer places a substantial burden on both the health and economy of individuals.

The incidence of breast cancer remains persistently high; however, the 5-year overall survival rate for female breast cancer has reached 90% [2], primarily attributed to continuous advancements in early diagnosis and comprehensive treatment strategies for breast cancer [3]. Traditional diagnostic tools for breast cancer, such as clinical and physical examinations, histopathology, ultrasound, magnetic resonance imaging (MRI), cytology, and biopsies [4,5], as well as the application of various biosensors based on principles like atomic force microscopy, Raman spectroscopy, electrochemical spectroscopy, microfluidics, and fluorescence [6,7,8], have played a crucial role in early breast cancer diagnosis. Furthermore, the use of treatments such as surgery, chemotherapy, radiation therapy, and endocrine-targeted therapies, including tamoxifen, aromatase inhibitors, and trastuzumab [9,10,11], has ensured the potential for breast cancer cures. Despite technological innovations and improvements in treatment modalities leading to significant clinical progress, breast cancer’s strong invasiveness and high susceptibility to recurrence and metastasis remain as significant challenges affecting clinical prognosis [2]. 

Breast cancer metastasis is the leading cause of patient mortality, and it is estimated that around 90% of breast cancer-related deaths are attributed to cancer cell metastasis [12]. The metastasis of breast cancer is a long and complex process involving mutations or changes in the expression levels of multiple key genes, as well as the activation or inhibition of related pathways. Previous studies have shown that the molecular alterations in primary tumors differ from those in metastatic breast cancer. For example, metastatic breast cancer typically exhibits more molecular biomarker alterations, such as increased expression of MET, EGFR, and PD-L1, which may lead to resistance to specific targeted therapies [13,14,15]. Additionally, metastatic breast cancer may manifest a greater inclination toward epithelial–mesenchymal transition (EMT), a molecular characteristic that enhances the invasiveness of cancer cells, with EMT markers such as N-Cadherin and Vimentin typically upregulated in metastatic tumors [16,17]. Therefore, in addition to the early diagnosis of primary breast cancer, new methods are still required for the early diagnosis of metastatic breast cancer, aimed at guiding the treatment of metastatic disease. 

To prevent the recurrence of breast cancer and enhance the overall survival rate of breast cancer patients, early warnings of breast cancer metastasis are a meaningful endeavor. Currently, commonly used techniques such as MRI, bone scanning, breast and axillary ultrasound, chest CT, the detection of tumor markers CA 15-3, CA 27.29, and CEA, lymph node biopsy, and other methods can be used to determine the presence of breast cancer metastasis [18,19]. The existing techniques and methods are often effective, but typically invasive, expensive, time-consuming, and require laboratories with advanced infrastructure for implementation. Therefore, biosensors have been extensively developed and applied in the early warning of breast cancer metastasis due to their straightforward, low-risk nature, analytical specificity, cost-effectiveness, and high sensitivity [20,21,22].

Recently, there have been numerous reviews on breast cancer, offering detailed descriptions of biosensors for breast cancer diagnosis and biomarkers in the blood of metastatic breast cancer patients [23,24,25,26,27,28]. Nevertheless, a comprehensive review on biosensors for early warnings of metastatic breast cancer is still missing. Based on a comprehensive literature review, this review first describes the mechanisms and key biomarkers in the development of breast cancer. It then focuses on different types of biosensors for the recognition of key markers during the breast cancer metastasis process, including electrochemical, optical, and microfluidic chip-based sensors, as well as quartz crystal microbalance (QCM) sensors, and discusses their individual performances, advantages, and limitations. Finally, the challenges in the clinical implementation of biosensors are discussed, along with the future prospects of biosensors.

## 2. Research Methodology

This study constitutes, methodologically, an analytical bibliographic review related to the preparation of different types of biosensors involved in the detection of metastatic breast cancer, and elaborates on the specific role of different sensors in the early warning of breast cancer metastasis. Data collection was carried out from May 2023 to July 2023, using the following databases: PubMed, Science Direct from Elsevier, the Wiley Online Library and Springer Nature, ACS—American Chemical Society, and Google Scholar, as well as databases of scientific articles and patents, “The LENS” and “ORBIT Intelligence”.

The inclusion criteria for this work included original articles exclusive to the metastatic breast cancer and biosensors studied, with full texts available in Portuguese, English, and other languages. The exclusion criteria included abstracts, online sites without scientific sources, incomplete texts, and unrelated and repeated articles.

Regarding search strategies, the descriptive terms used in this work are as follows: (breast cancer, breast cancer tumor, breast cancer tumor, breast cancer lymphedema) AND (transfer, transfer, transfer) AND (sensors, detection). The articles were selected by reading the titles and abstracts of the publications, associated with the Boolean descriptor “OR” and “AND”, in order to refine the samples.

**Figure 1:**
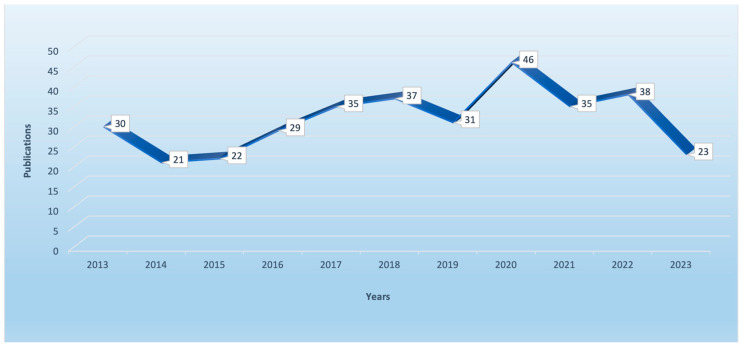
Number of publications related to the topic of this article and patents in the last 10 years, using the Web of Science as a database (keywords “biosensor” and “breast cancer metastasis”).

This review is based primarily on articles published after 2013, as seen from Figure 1. However, some older articles were also mentioned to provide relevant background or when providing well-documented information. This study shows that the potential and development of different types of biosensor preparation involving the detection of metastatic breast cancer is worth looking into.

## 3. Metastatic Breast Cancer and Related Biomarkers

Breast cancer is a highly heterogeneous malignant tumor characterized by the uncontrolled proliferation of breast epithelial cells under the influence of multiple carcinogenic factors [2].

### 3.1. Breast Cancer

In 2011, the International Breast Cancer Association classified breast cancer into four subtypes based on the expression of three tumor markers: estrogen receptor (ER), progesterone receptor (PR), Ki-67, and human epidermal growth factor receptor 2 (HER2). These subtypes include Luminal A, Luminal B, HER2 overexpression, and triple-negative breast cancer (TNBC) [29]. The molecular subtyping of breast cancer is closely associated with breast cancer metastasis. Among these, TNBC exhibits the highest invasiveness, with approximately 50% of TNBC patients experiencing distant metastasis [30]. The postoperative recurrence rate can reach as high as 25%, and the mortality rate within 3 months after recurrence is as high as 75% [31]. Furthermore, Luminal A breast cancer has the best prognosis, but due to its higher incidence, the number of cases with metastatic recurrence is significantly greater than other subtypes [32].

### 3.2. Breast Cancer Metastasis

Breast cancer metastasis involves the dissemination of cancer cells from the primary tumor site to other areas of the body, including the lymph nodes, lungs, bones, and the liver, among others [12]. Currently, our understanding of the molecular mechanisms involved in the process of tumor metastasis is very limited. The “seed and soil” hypothesis, introduced by the British surgeon Stephen Paget in 1889 [33], is likely the most suitable definition for this phenomenon. Based on this theory, the process of breast cancer metastasis can be simplified into the following steps: Malignant tumor cells detach from the primary breast tumor and enter the bloodstream or lymphatic system, then spread through these internal channels to other parts of the body, such as the lymph nodes, lungs, bones, liver, and so on. Within the target organs, these cancer cells undergo a series of complex interactions, including adhesion, invasion, establishment, and proliferation, ultimately forming secondary lesions [34]. At present, it has been established that breast cancer metastasis is a complex multi-step process involving factors such as cell gene mutation, tumor microenvironment changes, and angiogenesis, among which functional biomolecules such as proteins, nucleic acids, and metabolic small molecules can objectively and specifically reflect the occurrence and development of tumors [19]. Detecting biomarkers in this process forms the basis for the early identification of metastatic breast cancer.

### 3.3. Breast Cancer Metastasis Markers Suitable as Targets of Biosensors

Biomarkers are typically defined as “features that can be objectively measured and evaluated as indicators of normal biological processes, pathological processes, or pharmacological responses to therapeutic interventions” [35]. Biomarkers can exist within cells or outside of cells, and quantifying their changes can be used to distinguish between healthy individuals and cancer patients, as well as to indicate the progression of breast cancer. As seen from Table 1, the potential biomarkers that can be used as biosensors include cell surface receptor proteins, mutated genes, microRNAs, cells, exosomes, etc.

#### 3.3.1. Glycoprotein

Cell surface glycans play a crucial role in various biological processes such as intercellular communication, immunity, infection, development, and differentiation. MUC 1 is a transmembrane mucin glycoprotein expressed in most epithelial tissues, and research has shown its association with breast cancer cell adhesion, immunity, and metastasis, making it a valuable biomarker for monitoring breast cancer metastasis [36]. Additionally, in metastatic breast cancer, CEAs and CA15-3 can be used to distinguish bone metastases from other metastatic lesions [37]. CA15-3 and TPS levels significantly increase in liver metastasis patients. When TPS levels are normal and other tumor markers are elevated, the suspicion of lung metastasis may arise [38]. Similar to CA 15-3, CA 27.29 is also a marker used for monitoring breast cancer progression and treatment response. It can elevate during the process of breast cancer metastasis [39]. Lastly, CEAs (carcinoembryonic antigens), as non-specific tumor markers, may also increase in some breast cancer patients, especially when cancer cells have metastasized to other sites [40].

#### 3.3.2. Nucleic Acid

The rate of DNA release from metastatic tumor cells is higher than that of normal cells. Many DNA molecules have been found to play a crucial role in the occurrence and metastasis of breast cancer. For example, the amplification of the HER2 gene can lead to the rapid growth and spread of cancer cells, and the expression of BRCA1, BRCA2, EGFR, and PIK3CA can also increase the proliferation and migration risk of cancer cells [41]. Furthermore, circulating free DNA is a type of extracellular DNA in plasma or serum, and its quantity is significantly correlated with patients with metastatic breast cancer, serving as a biomarker for metastatic breast cancer tumors [42]. 

MicroRNAs are short RNA molecules that regulate gene expression and play an important role in the development and metastasis of breast cancer. For example, the high expression of miR-10b can promote the invasive ability of tumor cells into other tissues [43]. The miR-200 family (miR-200a, miR-200b, miR-200c, miR-141, and miR-429) is believed to be associated with the suppression of breast cancer metastasis because they can inhibit EMT, which is a process related to the invasion and metastasis of tumor cells [44].

#### 3.3.3. Circulating Tumor Cells

Apart from nucleic acids and proteins as biological molecules, tumor cells themselves are also considered as a type of tumor marker. When breast cancer cells detach from the primary tumor and enter the bloodstream, they are termed circulating tumor cells (CTCs). There is growing interest in CTCs as markers for breast cancer metastasis [45]. By detecting CTCs levels, staging and grading can be performed for patients with metastatic breast cancer. Furthermore, most CTC detection methods target the expression of epithelial-specific markers, such as the overexpression of CSV, N-Cadherin, and Twist, as well as the decreased expression of epithelial markers EpCAM, CK, and E-Cadherin [46].

#### 3.3.4. Others

In addition to nucleic acids, proteins, and cellular tumor markers, there are other tumor markers that play an important role in revealing the process of breast cancer metastasis, such as exosomes. Exosomes are small membrane-bound vesicles containing a wide range of molecules, including proteins, DNA fragments, miRNA, and lipids [47,48]. Exosomes mediate communication between cancer cells and normal or cancer-associated stromal cells to regulate tumor growth and metastasis. For example, exosomes derived from astrocytes can transfer PTEN (Phosphatase and Tensin Homolog)-targeting miRNA to tumor cells to suppress PTEN expression and promote metastasis [49]. Tumor-derived exosomes are gradually emerging as ideal biomarkers in the breast cancer metastasis process.

## 4. Biosensors for Detection of Metastatic Breast Cancer

The detection of breast cancer metastasis is of utmost importance for patient prognosis. Traditionally, the detection of metastatic breast cancer has relied on techniques such as computed tomography (CT), X-rays, positron emission tomography (PET), and nuclear magnetic resonance imaging (NMRI) [50]. Although the existing techniques and methods are viable, they tend to be invasive, costly, time-consuming, and necessitate laboratory facilities with advanced infrastructure for execution. Therefore, the development of a rapid and sensitive sensor for detecting relevant biomolecules in the process of tumor metastasis holds practical significance [51,52].

**Figure 2:**
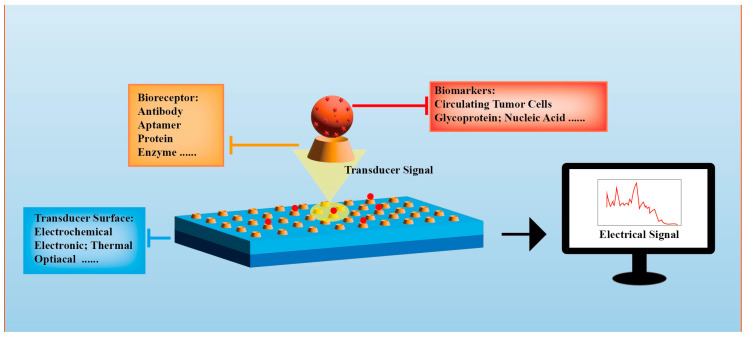
Basic structure of biosensors. Adapted from Ref. [54].

The structure of biosensors typically comprises several key components, as seen from Figure 2. Recognition Element [53,54]: This is the core component of biosensors used to selectively identify target biomolecules. The recognition element is typically a biomolecule such as antibodies, enzymes, nucleic acid probes, or cell receptors, possessing specificity for binding to the target molecule. It is a crucial part of biosensors and can determine their functionality and performance. Transducer: The transducer connects the recognition element with the signal processing system, often employing methods like optics, electrochemistry, or acoustics. It is responsible for converting recognition events into measurable physical signals. A strong coupling between the biomolecular recognition element and the transducer is essential for achieving the high selectivity of biomolecules and efficient signal conversion. Signal Processing System: this serves the purpose of processing, recording, and displaying data for researchers in a convenient format, whether analog or digital.

### 4.1. Electrochemical Biosensors

Electrochemical biosensors are devices that investigate the electrochemical behavior of electrically active surfaces to generate quantitative or semi-quantitative information using electrochemical transducers. Electrochemical readings are primarily obtained through techniques such as cyclic voltammetry (CV), differential pulse voltammetry (DPV), square wave voltammetry (SWV), electrochemical impedance spectroscopy (EIS), amperometry-based biosensors, and organic electrochemical transistors (OECTs), and they have been widely utilized in early-stage metastatic breast cancer due to their attributes of high sensitivity, specificity, portability, user-friendliness, and rapid response [51,52,55,56,57,58].

#### 4.1.1. Cyclic Voltammetry-Based Biosensors

CV is an electrochemical technique based on potential scanning used for detecting the concentration or activity of biomolecules. CV relies on the interactions between biomolecules (such as proteins, DNA, enzymes, etc.) and the modified layer on the electrode surface. The fundamental principle of CV entails applying a controllable potential (voltage) to the working electrode and scanning the potential linearly or nonlinearly at a certain rate [59]. Inferences about the concentration of target biomolecules or insights into the reaction mechanism are drawn from observations of current changes.

Elevation platelet-derived growth factor (PDGF-BB) concentrations in plasma could indicate the accelerating growth of metastatic breast tumors and angiogenesis. Mohammad et al. [55] immobilized a highly specific DNA aptamer for PDGF-BB onto gold nanoparticles loaded with α-cyclodextrin and further modified it for the detection of PDGF using SWV and CV techniques. Under optimized conditions, the calibration curve for PDGF-BB exhibited linearity within the range of 0.52–1.52 nM, with a quantitative limit of 0.52 nM, demonstrating the excellent analytical performance of the sensor. Satisfactory results were also obtained in tests of its selectivity, stability, and repeatability.

#### 4.1.2. Differential Pulse Voltammetry-Based Biosensors

DPV is an electrochemical sensor technology that achieves highly sensitive and specific electrochemical analysis by applying pulse potentials to the electrode and measuring the corresponding current response. Its distinctive feature is the reduction of background noise and enhancement of sensitivity through differential measurements [60].

As valuable biomarkers for cancer metastasis, the detection of CTCs helps to elucidate trends in the quantity and types of cancer cells. Shen et al. [51] successfully established an ultra-sensitive electrochemical aptamer sensor for CTCs based on DPV. It can detect CTCs in the range of 2 to 2 × 10 cells per milliliter with a limit of detection (LOD) of 1 cell per milliliter. Furthermore, the aptamer sensor can accurately identify CTCs in a mixture of various tumor cells, providing a basis for cancer progression assessment through clinical blood sample analysis. Importantly, the aptamer sensor can highly selectively isolate captured CTCs without compromising their viability. Therefore, the developed aptamer sensor not only holds great potential for early CTC detection but also contributes to individualized research at the molecular and cellular levels. Mahdi et al. [61] developed an electrochemical aptamer-based nanobiosensor with high sensitivity and selectivity for a quantitative and qualitative measurement of the HER2-ECD oncomarker. They evaluated different essential techniques utilized in the development of biosensors such as EIS, DPV, and CV. Finally, they chose the DPV method to detect the electrochemical signal. The GE-based aptasensor had a noteworthy and conducive result against HER2-ECD with a wide dynamic range of 10.0–500.0 ng/mL, a low limit of detection (LOD) of 0.667 ng/mL (significantly less than the clinical cut-off), and a low limit of quantification (LOQ) of 2.01 ng/mL. The results of this study confirmed that this promising electrochemical aptasensor could be feasibly applied as a platform for the diagnosis and monitoring of a wide variety of oncomarkers in different cancers. Sadeghi et al. [62] constructed a novel and well-organized g-aptasensor for detecting and determining various cancer cells, with a linear dynamic range of 5.0 to 10.0 × 10^4^ cells/mL, an analytical limit of detection (LOD) as low as 1.0 cell/mL, and a limit of quantification (LOQ) of 3.0 cells/mL. The advantages of this highly efficient nanocomposite platform include a broad dynamic range, high specificity, selectivity, stability, reproducibility, and low cost.

#### 4.1.3. Square Wave Voltammetry-Based Biosensors

SWV is a technique where a series of forward and reverse pulse signals (each with a consistent duration and applied at a specific frequency) are overlaid on top of a potentiator’s staircase linear scan. The subtraction of current between the forward and reverse pulses results in a differential current curve, enhancing measurement sensitivity [63]. 

Based on the role of CTCs in the diagnosis of metastatic breast cancer, Shen et al. [52] combined the electrochemical current generated by DNA with rolling circle amplification (RCA) and magnetic nanoparticles for the efficient capture and ultra-sensitive detection of CTCs. The researchers first used magnetic nanoparticles modified with antibodies against epithelial cell adhesion molecules (EpCAMs) to capture and enrich CTCs. Subsequently, nucleic acid aptamers were bound to the surface of CTCs, and subsequent RCA assembled a large number of DNA molecules on the electrode. Then, the reaction between DNA molecules and molybdic acid formed molybdenum phosphate via redox and generated an electrochemical current. Finally, the detection of various concentrations of MCF-7 was measured using SWV, allowing for the detection of MCF-7 cells within the range of 5 to 3 × 10^4^ cells per milliliter, with an LOD of one cell per milliliter.

#### 4.1.4. Amperometry-Based Biosensors

Amperometry is based on the principles of Faraday’s electrochemistry, where a specific reaction occurs between biomolecules and the biorecognition elements immobilized on an electrode upon the application of a certain electrical potential (voltage), leading to a change in current. This current change is associated with the presence, concentration, or activity of biomolecules [64]. 

Hypoxia-inducible factor-1α (HIF-1α) is a transcription factor that participates in tumor growth and metastasis by regulating genes involved in the hypoxia response. Cristina et al. [56] reported an electrochemical immunoassay based on magnetic beads (MBs) for the detection of HIF-1α. Researchers employed an MB-based sandwich immunoassay and amperometric detection using disposable screen-printed carbon electrodes (SPCEs), achieving highly sensitive detection with an LOD of 76 pg mL^−1^. This method was applied to the determination of cancer cells cultured in hypoxic conditions and saliva samples.

#### 4.1.5. Electrochemical Impedance Spectroscopy-Based Biosensors

EIS biosensors acquire information by measuring the complex impedance response of alternating current signals in electrolyte solutions at different frequencies. When biomolecules interact with the bio-recognition elements on the sensor surface, they have the potential to modify the complex impedance of the electrochemical system, and this alteration can be employed for the detection and analysis of target molecules [65]. 

The intravasation of metastatic cells into the blood stream, initiated by their invasion to vascular layers, would be a significant characteristic of metastasis. Human umbilical vein endothelial cells (HUVECs) would contract and disengage when attacked by metastatic cells. Based on the above principles, Mohammad et al. [57] developed an electrical biosensor based on Nano roughened PMMA that can record dramatic changes in the electrical response between metastatic and primary cancer cell interactions. Metastatic cells invaded the confluent endothelial barrier, retracting them from the surface. The penetration of the current from the perturbed endothelial layer observably reduced the impedance of the sensor. In contrast, primary cancerous cells could not invade to the HUVEC layer and no noticeable changes were observed in the impedance of the sensor. The response of each HUVEC-covered sensing well to the presence of primary or metastatic breast cancerous cells after known intervals of time were measured. Noticeable changes in the impedance (about 65%) were observed just 4 h after the interaction of rare metastatic breast cells with the endothelial barrier (with a concentration of 1:10). 

In addition, Wang et al. [58] developed an electrochemical cell sensor based on a multivalent aptamer nanostructure for the efficient detection of CTCs. The sensor not only demonstrates high sensitivity in detecting CTCs (with an LOD of 6 cells/mL in buffer solution) but also allows for further downstream analysis with high vitality upon release. This highlights the potential of EIS for early cancer metastasis diagnosis. Bi, L. et al. [66] proposed an effective apt sensing strategy using a halloysite nanotube/carbon composite decorated with Pd nanoparticles (HNT/C@Pd NPs) as a modifier to determine HER2 tumor markers using the electrochemical impedance spectroscopy (EIS) method. With a correlation coefficient of 0.996, the electrochemical apt sensor demonstrated a wide linear range from 0.03 ng/mL to 9 ng/mL. The limit of detection (LOD) of the established assay was 8 pg/mL based on the S/N = 3 method. This confirmed that the proposed methodology can be used in the quantification of breast cancer markers for early diagnosis and treatment.

The use of aptamers as receptors in electrochemical biosensors has increased sharply. Nanomaterials can be used as substrate for the immobilization of aptamers, or as labels to amplify electrochemical currents. Both factors improve detection limits. Rostamabadi et al. [67] presented a method for the electrochemical determination of the breast cancer biomarker, HER2. The assay is highly reproducible and specific, with a low limit of detection (50 fg·mL^−1^) and a wide analytical range (0.1 pg·mL^−1^ to 1 ng·mL^−1^).

#### 4.1.6. Organic Electrochemical Transistor-Based Biosensors

OECTs are a novel type of biosensor with wide-ranging applications. OECT sensors are based on the electronic conduction properties of organic semiconductor materials, where biological recognition elements are immobilized on the material’s surface. When target biomolecules interact specifically with the recognition elements, they can alter the electronic transport properties of the organic semiconductor, allowing for the detection and analysis of the presence, concentration, or other relevant information of the biomolecules through changes in current or conductivity [68,69]. 

Currently, research has reported the use of organic electrochemical transistor sensors to detect markers of metastatic breast cancer. Cell surface glycans play a critical role in various biological processes such as intercellular communication, immunity, infection, development, and differentiation. Chen et al. [70], based on the interaction between Con A immobilized on the gate electrode and cell surface mannose sites, successfully utilized OECTs to analyze mannose expression on live cancer cells. Due to the high expression of mannose on the cell surface, this device can selectively detect cancer cells at concentrations as low as 10 cells/μL.

As seen from Table 2, electrochemical biosensors offer high sensitivity and specificity in tumor detection, enabling the real-time monitoring of target biomarkers, and typically obviating the need for invasive sampling procedures. However, they are also influenced by sample complexity, and some samples require time-consuming pre-processing, necessitating the further optimization of the synthesis steps [57,70,71].

### 4.2. Optical Biosensor

Compared to traditional analytical techniques, optical biosensors offer significant advantages, allowing for the real-time and label-free detection of biomarkers with high sensitivity and specificity. The principle relies on the interaction between biomolecules and light to achieve the detection of biological molecules [72]. Typically, biosensors have biorecognition elements immobilized on their surfaces or detectors, such as antibodies, DNA probes, or fluorescently labeled biomolecules. When these biorecognition elements bind to the target molecules, optical signals, such as absorbance, fluorescence, scattering, or refraction changes, are generated, and these alterations can be measured and interpreted, thus enabling the high-sensitivity and high-specificity detection of biological molecules. Currently, optical-based biosensors have been developed for diagnosing various types of biomarkers. In principle, these biosensors operate by absorbing light from a light source and emitting optical signals. As seen from Table 3, they can be categorized into surface-enhanced Raman scattering (SERS), surface plasmon resonance (SPR), fluorescence, and chemiluminescence methods [73,74,75].

#### 4.2.1. Surface Plasmon Resonance-Based Biosensors

SPR is based on the phenomenon of plasmon resonance on a metal surface. When a light beam interacts with the metal surface, it induces plasmonic oscillations. Interactions between biomolecules and biorecognition elements immobilized on the metal surface lead to changes in surface plasmon resonance conditions. By recording optical images at different points, the real-time monitoring of the binding, distribution, and concentration of biomolecules is achieved, enabling the multi-channel, real-time detection of biomolecules [78]. 

In this category, to achieve a low concentration detection of CTCs, Médéric et al. [73] presented the use of specific aptamers directed against mammaglobin proteins, located at the surface of circulating breast cancer cells. Optical fiber grating sensors were functionalized using aptamer receptors to detect cells at low concentrations in vitro, and detection reaching 100 cells/mL was achieved with a label-free detection strategy (calculated LOD of 49 cells/mL), while the detection of only 10 cells/mL was observed using gold nanoparticles as a signal amplification tool. Similarly, in order to solve the problem of insufficient blood samples and low CTC concentration levels for current in vitro blood testing, Zhu et al. [74] developed an intravenous surface plasmon resonance fiber probe capable of real-time CTC detection in blood. By exposing protein-functionalized fiber probes to circulating blood, the continuous capture of CTCs ensures a constant enrichment, significantly enhancing cell counting accuracy. Further, a detection limit of ~1.4 cells per microliter was achieved by using an epithelial cell adhesion molecule (EpCAM) antibody-based receptor layer and a 15 min enrichment period. Many studies have shown that urokinase plasminogen activator (uPA) is causally involved in promoting cancer metastasis. Bipin et al. [75] developed a novel and scalable uPA sensor based on a graphene–gold nanoparticle platform that uses the fluorescence of quantum dots to rapidly (<1 h) detect uPA up to 100 pM. The work was based on graphene’s SPR and fluorescence quenching for the highly selective sensing of uPA, and has demonstrated its highly reliable, inexpensive, and easily scalable properties by detecting fluorescence intensity.

#### 4.2.2. Surface-Enhanced Raman Spectroscopy-Based Biosensors

The fundamental principle of surface-enhanced Raman spectroscopy lies in the utilization of nanoscale metallic structures, such as silver or gold nanoparticles, to generate a strong localized electric field enhancement effect. When laser light is irradiated onto samples containing target molecules, these metallic structures not only induce Raman scattering but also significantly enhance the scattered spectral signal through electromagnetic field enhancement, enabling the highly sensitive and specific detection of trace molecules [79]. In order to achieve better separation and detection of CTCs, Kunnumpurathu et al. [76] developed a SERS tag-enabled lab-on-a-filter system built over a custom-designed portable centrifugal prototype. The SERS nanotag (Au-rGO@antiErbB2) present in the system performed the accurate detection of CTCs, which played a key role in the isolation and quantification of CTCs among the millions and millions of healthy cells in the human bloodstream.

#### 4.2.3. Electrochemiluminescence-Based Biosensors

The principle of ECL biosensors involves the use of biological recognition elements, such as antibodies or DNA probes, which, by binding to the target biomolecules, excite fluorescently labeled biomolecules through electrochemical reactions or changes in potential, resulting in fluorescence signals. These fluorescence signals can be measured and analyzed, enabling the highly specific and sensitive detection of biomolecules [80]. 

The ultrasensitive monitoring of cancer cells, especially metastatic ones, has a great interest in human medicine. Despite the early diagnosis of diseases, there is an essential need for any prediction in the severity of side effects for therapeutic outcomes like metastasis. Therefore, Hassan et al. [77] developed an electrochemiluminescence (ECL)-based cyto-sensor for the quantification of metastatic breast cancer cells (SKBR-3). In this protocol, a silica-based electrode was prepared via in situ electrosynthesis of mesoporous silica; luminol (as luminophore) was combined with chitosan (as attachment biomolecule) to produce a stable lumino-composite film on the electrode surface. At the optimum experimental conditions, the lower limit of quantitation (LLOQ) and linear dynamic range (LDR) were obtained as 20 cells/mL and 20 to 2000 cells/mL.

### 4.3. Microfluidic Biosensors

**Figure 3:**
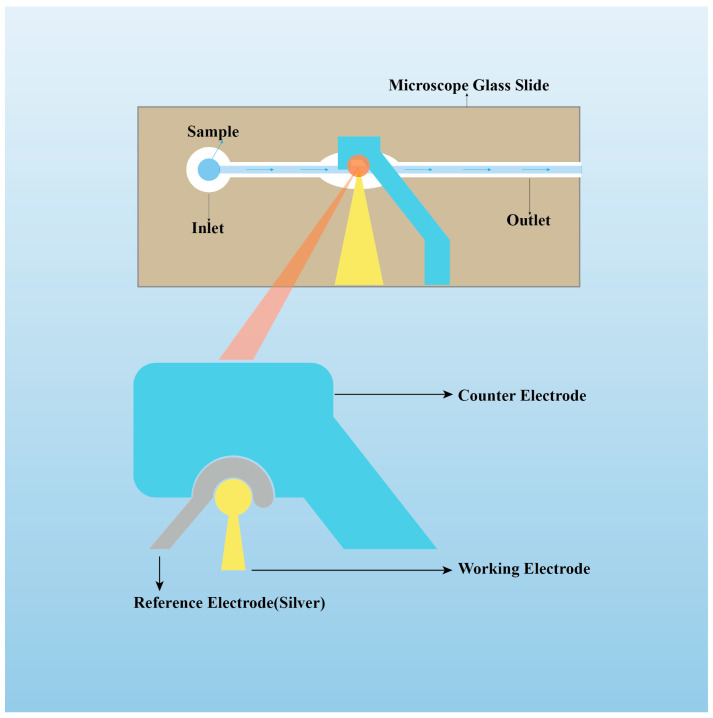
Device construction of microfluidic impedance biosensor chips for label-free detection of proteins. Adapted from Ref. [82].

Microfluidic biosensors refer to devices constructed with microchannels on a chip and modified with disease-specific antibodies to detect target analytes. Compared to traditional laboratory analysis methods, microfluidic technology offers advantages such as lower sample consumption, rapid analysis, high automation, miniaturization, and ease of integration. Its strong integration capabilities, in particular, enable the integration of various experimental processes, including reactions, pre-processing, and detection, into a single microfluidic system [81,82] (Figure 3). 

The detection of exosomes and other circulating cancer biomarkers in liquid biopsies is emerging as a new paradigm for non-invasive cancer diagnosis and prognosis monitoring. However, most microfluidic chip technologies available for analyzing exosomes primarily rely on antibody immunobinding or gene detection after extraction processes. Immunoassay methods highly depend on the binding affinity of receptors (such as antibodies) and require additional detection for confirmation. To address this issue, Jaewoo et al. [83] developed an exosome mRNA sensor based on a microfluidic chip (exoNA sensor) (Table 4). This sensor enables the one-step detection of exosome ERBB3 in blood by integrating a microfluidic chip and 2D nanostructure hydrogel. The sensing part of the sensor includes a 3D nanostructure hydrogel, which can amplify the fluorescence signal through an enzyme-free catalytic hairpin assembly reaction at room temperature to detect ERBB2 and reference genes, with a detection limit of 58.3 fM.

Furthermore, the detection of CTCs for assessing breast cancer metastasis has been validated through various methods; however, more sensitive methods are still needed. Tiberiu A. Burinaru et al. [84] developed a label-free detection method based on a microfluidic device, which is integrated with an EIS biosensor capable of capturing and quantifying CTCs. The researchers functionalized fork-shaped gold electrodes encapsulated in PDMS with specific antibodies, including anti-EpCAM and anti-CD36 antibodies, achieving high sensitivity for the detection of as few as 3 MCF-7 cells.

### 4.4. Others

In addition to electrochemical, optical biosensors, and microfluidic sensors [65,73,83], there are several other sensors applied in monitoring metastatic breast cancer, for example, QCM biosensors and metamaterial biosensors.

The principle of QCM biosensors is based on the resonance frequency changes caused by the tiny mass variations on the quartz chip’s surface due to processes like protonation, biomolecule adsorption, or biological reactions, enabling the highly sensitive detection of biomolecules, bio-interactions, or biological processes [87,88]. 

The HER2/neu receptor is typically overexpressed on the surface of highly metastatic breast cancer cells, and its levels can be indicative of the risk of breast cancer metastasis. Merve et al. [85] have developed a system based on specific and straightforward QCM to identify breast cancer cells expressing HER2/neu through receptor-specific monoclonal antibodies. Initially, scientists coated the QCM chip with polymer nanoparticles composed of hydroxyethyl methacrylate (HEMA) and ethylene glycol dimethacrylate (EDMA). Subsequently, they functionalized the QCM chip with nanoparticles by binding HER2/neu antibodies, achieving a detection limit of 10 cells/mL.

Metamaterials are artificial materials composed of periodically arranged sub-wavelength structures. They exhibit unique properties that many natural materials lack, garnering significant attention in recent years [89,90,91]. Particularly, metamaterials exhibit resonance absorption peaks when exposed to electromagnetic waves and are highly sensitive to changes in their surface dielectric environment. Additionally, terahertz radiation possesses relatively low photon energy, effectively avoiding the harmful ionization of biological molecules, making it well-suited for biosensing. Therefore, metamaterials have been employed in protein, cell, and ribonucleic acid (RNA) sensing [92,93]. 

Metamaterial-based biosensors have been widely used for identifying cell types and detecting the concentration of tumor biomarkers. However, non-invasive in situ measurement methods for cell migration, which plays a crucial role in tumor progression and metastasis, are valuable for research. Fang et al. [86] developed a flexible terahertz metamaterial biosensor based on a polyethylene terephthalate substrate for the label-free and non-destructive detection of breast cancer cell growth and migration (Table 4). The biosensor monitored the growth behavior of breast cancer cell MDA-MB-231 by introducing a PDMS barrier sheet as a “wound”. Furthermore, the effects of TGF-β on cell migration were investigated on the surface of the metamaterial biosensor, and demonstrated that the migration of MDA-MB-231 would be significantly enhanced when increasing the concentration of TGF-β. Therefore, metamaterial biosensors provide a new approach for detecting cell growth and migration, showing significant potential in future cancer cell biology and biomedical research.

## 5. Conclusions and Future Outlook

Breast cancer metastasis profoundly impacts patients’ physical health and quality of life, making the early detection of breast cancer metastasis crucial for disease management. Currently, research on biosensors for detecting breast cancer metastasis has made a significant progress, leading to the emergence of electrochemical and optical biosensors, microfluidics, and novel sensor types. Among various types of biosensors, electrochemical and optical biosensors have consistently been predominant. Although they are composed of different molecular recognition elements and sensors, their common characteristics include high sensitivity, high accuracy, relatively short detection times, and ease of use compared to traditional detection methods. Advances in understanding the mechanisms of breast cancer metastasis, coupled with the emergence of new biosensor technologies and novel materials, have propelled further development in the field of biosensors. For instance, microfluidic detection technology has greatly enhanced the convenience of detection [83], QCM sensors offer the ability to obtain abundant real-time online information [85], and the emergence of metamaterials provides possibilities for the non-destructive detection of biomarkers [86].

The primary challenge in the development of biosensors lies in the selection of recognition elements. Due to an incomplete and in-depth understanding of breast cancer metastasis mechanisms, the discovery of representative biomarkers for the breast cancer metastasis process is still in its early stages. While some typical biomarkers have been proven to be closely associated with breast cancer metastasis, more biomarkers need to be discovered. Improving the study of biomarkers and elucidating the underlying mechanisms linking these biomarkers to disease status and breast cancer progression can facilitate their better application in sensors. In particular, it should be noted that the application of circulating cells as markers in the diagnosis of metastatic breast cancer is increasing; in essence, it is also the use of specific targets on circulating cells to identify. Although markers in circulating cells are still far from the real practical application, we expect more applications based on circulating cell biosensors in metastatic breast cancer. Furthermore, most current biosensors rely on the detection of a single biomarker, lacking the ability to simultaneously detect different types of targets. Using a single biomarker alone does not provide clinicians with comprehensive information about cancer progression, as factors like patient treatment and status are also related to cancer metastasis.

Most biosensors lack biocompatibility in their fixed matrices, leading to numerous non-specific bindings when exposed to serum or real patient samples. The decreased affinity between sensors and biomarkers poses a significant challenge in biosensor development. To overcome this issue, the primary approach is to search for stable materials to immobilize molecular recognition elements for the stable transmission of various biological signals. For instance, the development of novel graphene nanocomposites can enhance the stability of electrochemical sensors in high-ionic-strength environments [94]. Secondly, combining nanomaterials with signal amplification strategies can improve sensitivity and specificity. In summary, researching new materials or developing new strategies is a focal point in biosensor platform development.

In conclusion, various biosensors have demonstrated immense potential in the detection of breast cancer metastasis. However, most related research is currently at the basic research stage. The focus of future research should be on the early clinical application of biosensors, transitioning from basic research towards stronger stability, higher throughput, miniaturization, convenience, and commercialization.

## Figures and Tables

**Table 1:** Typical biomarkers for the detection of breast cancer metastasis.

Classification	Typical Biomarkers	Ref.
Glycoprotein	MUC 1; CEA; CA15-3; TPS; CA 27.29	[36,37,38,39,40]
Nucleic acid	HER2 gene; BRCA1; BRCA2; EGFR; PIK3CA; miR-10b; miR-200 family (miR-200a, miR-200b, miR-200c, miR-141, and miR-429)	[41,42,43,44]
Circulating tumor cells	CSV; N-Cadherin; Twist; EpCAM; CK; E-Cadherin	[45,46]
Others	Exosomes transfer PTEN-targeting miRNA	[47,48,49]

**Table 2:** Key characteristics of electrochemical biosensors for the detection of metastatic breast cancer.

Target Biomarker	Bare Electrode	Electrode Modification	Detection	LOD	LR	Year	Ref.
Mannose	Au	Carbon nanotubes.	Fluorescence	10 cells μL^−1^	None	2018	[70]
Elevation platelet-derived growth factor (PDGF-BB)	Au	One-step template (α-cyclodextrin)-assistant green electrodeposition method.	SWV and CV	0.52 nM/328 cells mL^−1^	0.52–1.52 nM/328 to 593 cells mL^−1^	2018	[55]
MCF7 and MDA-MB231	Au	Nanoroughened PMMA substrate.	EIS	None	None	2018	[57]
EpCAM of MCF-7	Au	Antiepithelial cell adhesion molecule (EpCAM) antibody-modified magnetic nanospheres.	CV and SWV	1 cells mL^−1^	5 × 10^0^ to 3 × 10^4^ cells mL^−1^	2019	[52]
Hypoxia-inducible factor-1 alpha (HIF-1α)	Carbon	The whole suspension of MBs modified with the sandwich immunocomplexes were pipetted on the working electrode surface of the SPCE.	Amperometric	76 pg mL^−1^	None	2020	[56]
Tetrahedral DNA nanostructures	Au	Tetrahedral DNA nanostructures labeled with thiol are immobilized onto the surface of SPGE by Au–S bonds.	CV	3 cells mL^−1^	None	2020	[71]
MUC1-targeting aptamer	CeO2@Ir nanorods	Binding of biotin- and carboxyl-modified D-RNA to magnetic beads (MBs) and Ce@IrNRs.	DPV	1 cell mL^−1^	2 to 2 × 10^6^ cells mL^−1^	2020	[51]
EpCAM; CSV	Au	Self-assembly product, TCEP, and DNA fixation buffer were dropped onto the electrode.	EIS	6 cells mL^−1^	None	2023	[58]
HER2-ECD oncomarker	Graphite electrode	Reduced graphene oxide nano-sheets (rGONs) and rhodium nanoparticles (Rh-NPs) on the graphite electrode (GE) surface.	DPV	0.667 ng/m	Dynamic range of 10.0–500.0 ng/mL	2022	[61]
HER2	Graphite	The hybrid nanocomposite established by the coupling of reduced graphene oxide nanosheets (rGONs) and rhodium nanoparticles (Rh-NPs) on the surface of graphite electrodes.	DPV	1.0 cell/mL	5.0 to 10.0 × 10^4^ cells/mL	2022	[62]
HER2	Halloysite nanotube/carbon composite	Halloysite nanotube/carbon composite decorated with Pd nanoparticles (HNT/C@Pd NPs) modifier.	EIS	8 pg/mL	0.03 ng/mL to 9 ng/mL	2023	[66]
HER2	Glassy carbon electrode	Glassy carbon electrode (GCE) was modified with densely packed gold nanoparticles placed on a composite consisting of electrochemically reduced graphene oxide and single-walled carbon nanotubes (ErGO-SWCNTs).	EIS	50 fg/mL	0.1 pg·/mL to 1 ng/mL	2019	[67]

**Table 3:** Key characteristics of optical biosensors for the detection of metastatic breast cancer.

Target Biomarker	Supporting Substrate	Modification	Detection Method	LOD	LR	Ref.
mammaglobin-A	Au	Optical fiber grating sensors were functionalized using aptamer receptors.	Surface plasmon resonance (SPR)	49 cells mL^−1^	None	[73]
EpCAM of MCF-7	Fiber probe	Au film and EpCAM (MCF-7) antibody were deposited.	Spectrometer	~1.4 cells uL^−1^	None	[74]
uPA (urokinase plasminogen activator)	Graphene	Modified with gold and antibodies.	Fluorescence	100 pM	None	[75]
EpCAM and ErbB2 of SKBR3 cells	Au	Antibody modification, Raman scattering enhancement.	SERS and fluorescence imaging	5 cells mL^−1^	None	[76]
HER-2 of SKBR-3	Silica	Luminol was combined with chitosan to produce a stable lumino-composite film on the electrode surface.	ECL; CV	20 cells mL^−1^	20 to 2000 cells/mL	[77]

**Table 4:** Key characteristics of microfluidic and other biosensors for the detection of metastatic breast cancer.

Type of Sensor	Target Biomarker	Supporting Substrate	Modification	Detection	LOD	Ref.
Microfluidic biosensors	ERBB2	None	None	Fluorescent signal	58.3 fM	[83]
	EpCAM and CD36 of MCF-7	Au	Functionalized with anti-EpCAM antibodies.	CV	None	[84]
Quartz crystal microbalance	HER2/neu	Au	Polyhydroxyethyl methacrylate nanoparticles changed the hydrophobic properties of the surface of the gold QCM chip.	QCM device	10 cells/ml	[85]
Terahertz metamaterial biosensor	Transform growth factor-β (TGF-β)	Silicon wafer	Au is deposited in Parylene by an electron beam and then coated with antibodies.	Fluorescent images, terahertz time domain spectroscopy.	None	[86]

## Data Availability

Not applicable.
